# Insights into self-reported food allergies in Romanian schoolchildren

**DOI:** 10.3389/falgy.2024.1472673

**Published:** 2025-01-21

**Authors:** Claudia Felicia Pop, Daniela Rajka, Ioana Corina Bocsan, Petronela Alina Coblisan, Gabriela Edita Ichim, Anna Lazar, Paraschiva Chereches-Panta

**Affiliations:** ^1^ Faculty of Nursing and Health Sciences, Iuliu Hatieganu University of Medicine and Pharmacy, Cluj-Napoca, Romania; ^2^ The Society of Doctors from Children's and Youth Collectivities, Medical School Office, Cluj-Napoca, Romania; ^3^ Department of Pharmacology, Toxicology and Clinical Pharmacology, Iuliu Hatieganu University of Medicine and Pharmacy, Cluj-Napoca, Romania; ^4^ Mother and Child Department, Third Pediatric Discipline, Faculty of Medicine, Iuliu Hatieganu University of Medicine and Pharmacy, Cluj-Napoca, Romania; ^5^ Regina Maria Hospital, Department of Obstetrics-Gynecology, Cluj-Napoca, Romania

**Keywords:** food allergy, schoolchildren, food-induced anaphylaxis, prevalence of food allergy, teachers

## Abstract

The prevalence of food allergy (FA) varies worldwide with an increasing rate in the last decades. Data of self-reported FA have been recorded by most European countries, the US, Canada and Australia, but not Romania. The aim of this study is to analyze the prevalence and severity of FA and to assess the extent of information the medical and teaching staff in schools have on students’ medical history.

**Methods:**

A cross-sectional survey was performed in schoolchildren from Cluj-Napoca, Romania, using an online questionnaire delivered to their parents.

**Results and conclusions:**

Seven hundred and eight individuals completed the entire questionnaire. The prevalence of self-reported FA was 8.9%, 28.6% presented food-induced angioedema and 38.1% required ER presentation. Cow milk (36.5%), egg (9.5%), strawberry (20.6%) and nuts (2.7%)were the most frequent culprit foods. The lack of an appropriate and accurate communication with the medical and teaching staff in the school suggest the requirement for further measures for parents and children educations regarding food allergy detection and management.

## 1 Introduction

Food allergy is still a major concern worldwide due to both the increasing prevalence of the disorder and its burden in the specific management. Schoolchildren, mainly those in their first decade of life, represent a vulnerable age category. Food allergy imposes a great burden on both patient and family, affecting their emotional status. The risk of a severe reaction induces anxiety in the daily life of children with FA and is often associated with significant limitations in their social interactions (1, 2).

The prevalence of food allergy (FA) varies according to many factors. Recently, Lyons SA et al. reported the prevalence of FA in schoolchildren aged 7–10 years, in a cross-sectional study performed in 8 European countries (1). The prevalence of self-reported FA varied between 13.1% and 47.5%, the lowest in Greece and the highest in Poland and Lithuania (1).

By the end of 2021, an interesting review on food allergy globally was published, focusing on incidence, diagnosis and therapy of FA in different guidelines (2). The authors pointed out the differences between different continents in the prevalence of self-reported FA and the relevance of the definition of FA (2).

Self-reported FA is an obvious cause for overestimated prevalence of FA. Nevertheless cross-sectional surveys on significant large population groups showed that self-reported FA estimated rate is extremely variable among countries and continents, varying from 5 to 6 up to 19% in some African countries (3). The confirmed FA based on oral food challenge test has a lower rates in most of the studies (3, 4). In USA survey showed that a prevalence of 7.6% probable IgE-mediated FA (38,408 parent-reported FA in a 2018 US survey) (4).

A suspicion of food-induced allergic reaction should be confirmed in order to have a positive diagnosis of food allergy. The gold standard for the diagnosis of food allergy is an oral double-blind placebo-controlled food challenge (DBPCFC) to the culprit allergen that elicits reproducible clinical symptoms (5). Because the DBPCFC may induce severe reactions, it is not used routinely in most clinical settings. Trained doctors, who are equipped to manage potential adverse reactions, including anaphylaxis (6), can underwent it only under close clinical observation. Other diagnostic tools, like skin prick tests with standardized extracts or culprit food, sIgE to whole extract or to components, where available, allow an accurate assessment of FA and they can also identify the patients that might need oral food challenge (OFCs) test. Thus, an extended analysis of the factors associated with the presence and severity of FA is necessary in order to help the physicians from schools to provide adequate care to schoolchildren and refer them to an allergy specialist.

The main aims of our study are to establish the lifetime prevalence of FA in schoolchildren in Cluj-Napoca, Romania, based on parents self-report, and to assess the level of information available to medical and teaching staff in the school about their students' medical history and their awareness of possible severe reactions. Secondary objectives of this study are to characterize the clinical features of FA, to identify possible risk factors for FA, to evaluate the correlation between FA and other allergic diseases in children and to assess the impact of it in the child's social relationships.

## 2 Materials and methods

This is an open cross-sectional non-randomized survey study. We conducted the study in March 2023 in four schools from Cluj-Napoca, Romania. The study protocol and the survey content were approved by the Ethics Committee of Iuliu Hatieganu University of Medicine and Pharmacy (no. AVZ62/2023).

### 2.1 Method

The study is based on an anonymous questionnaire delivered online to parents by the school doctor who works in the institution where the child studies. An informative letter addressed to the parents about the outcomes of the study, the anonymous and non-coercive nature of participation in the study, and the approval of the Ethics Committee were added to the questionnaire.

All primary and secondary school students were included in the study and their parents received the online questionnaire. A number of 708 parents (54.9% of referred questionnaires) completed the questionnaire entirely and data were included in the analysis.

All schoolchildren whose parents answered the questionnaire were included in the study. After analyzing the answers, the study participants were divided into 2 groups: group A, students with a history of food allergy, and group B, students with no history of food allergy. Parents reported FA based on convincing clinical history and prior diagnosis of FA established by a physician, either paediatrician or allergist.

### 2.2 Collected data

The information in the questionnaire refers to:

1. Demographic data: age (date of birth), gender, the child's level of education (primary school, between 0 and 4th grade, or lower secondary education, between 5th and 8th grade);
2. Data regarding food allergy: the age of onset for the first symptoms, clinical manifestations (hives, flexural eczema, angioedema, gastrointestinal manifestations), the culprit foods, dietary interventions and the history of previously required medication (H1 antihistamines, adrenaline, corticosteroids). The foods that were listed in the questionnaire were: milk or dairy products (yoghurt, cheese, butter), hen egg, peanut, tree nuts (hazelnut, walnut or other nuts), wheat or cereals, soybeans, fish and seafood, and also several types of non-priority food like citrus fruits, strawberries, kiwi, chocolate. We included an open-ended question in the list so that the parent could add other potential culprits besides the ones listed in the questionnaire.
3. Data regarding the child's medical history: previous diagnosis of allergic diseases (asthma, allergic rhinitis, allergic conjunctivitis) and their impact on their social life and relationships.
4. Possible associated risk factors (duration of breastfeeding, family history of any allergic disorders). The questionnaire contained detailed history of allergic diseases in both parents and siblings. The last part of the questionnaire referred to a diagnosis of food allergy, atopic dermatitis, urticaria, allergic rhinitis or rhino-conjunctivitis, drug allergy, or asthma in both the mother and the father, and to any other sister or brother.
5. The extent of the information the teacher or the school doctor have about the children's medical history, the current diet, the daily treatment or the need for an emergency kit that the child may require under certain circumstances.

### 2.3 Statistical analysis

The results were analysed using Excel, SPSS version 19 and MedCalc Statistical Software version 19.0.3. The prevalence of self-reported food allergy was calculated as a percentage from the total number of analyzed questionnaires. The severity was reported based on the clinical data mentioned: the number of any emergency visit due to the child`s allergy to certain foods, or the need for adrenaline therapy, or prior use of self-administered adrenaline or systemic corticosteroids during an allergic episode. These were also reported as percentage from the total number of children that were included for the analysis.

We analyzed the positive predictive value, negative predictive value, the specificity and sensitivity of several allergic comorbidities, like asthma, allergic rhinitis, and/or allergic rhino-conjunctivitis, comparing the two groups, A and B. We also analyzed the positive predictive value, negative predictive value, the specificity and sensitivity of some risk factors like the duration of breastfeeding, family history of allergies by comparing the two groups, A and B.

All the answers were analyzed and if for all of the questions related to personal and family history of allergies a positive answer was counted, positivity of them was considered and defined in the statistical analysis, regardless of their number (only one or more than one positive answer to these questions).

We compared the students in the two groups, A and B, and the differences were analyzed using the Mann-Whitney test, the χ^2^ test and the *t*-test. We used SPSS version 19 to analyze the correlation between different variables in group A and group B, with bivariate correlation and also student's *t*-test. The conventional thresholds of a *p* value below 0.005 for statistical significance, and the confidence interval of 95% were applied for data interpretation.

## 3 Results

### 3.1 Demographic characteristics of the study group

After sending the questionnaires, 708 were completed entirely and were available for analysis. The study group consisted of 362 female students (51.1%) and 346 male students (48.9%) between ages 6 and 15.

The 708 students were divided into two groups: group A, consisting of 63 children with self-reported food allergy, and group B, in which we included 645 children without self-reported food allergy. The overall prevalence of FA in our study group was 8.89%. Gender distribution and age distribution (children between 6 and 10 years old vs. children between 11 and 15 years old) were similar into the two subgroups. Demographic data are shown in Table 1.

**Table 1:** Demographics of the two study groups.

	Group A with food allergy (*n* = 63)	Group B without food allergy (*n* = 645)
Males, no (%)	29 (46.03%)	312 (48.37%)
Age, mean ± SD (years)	10.69 ± 2.83	9.98 ± 2.68
Age groups, no (%)		
- 6–10 years	27 (42.85%)	352 (54.57%)
- 11–15 years	36 (57.14%)	293 (45.42%)
Onset of any allergic reaction, no (%)		
- Below 2 years of age	29 (46.03%)	59 (9.15%)
- Between 2 years and 4 years	9 (14.29%)	48 (7.44%)
- Above 5 years of age	15 (23.81%)	64 (9.92%)
- Newer	10 (15.87%)	474 (78.49%)
Nationality, no (%)		
- Romanian	44 (69.84%)	404 (62.63%)
- Hungarian	18 (28.57%)	236 (36.58%)
- Polish	0	1 (0.15%)
- Arab-Greak	0	1 (0.15%)
- Israeli	0	1 (0.15%)
- Moldavian	0	1 (0.15%)

Additionally to food allergies, the questionnaire included information regarding respiratory or skin allergic diseases. In group A, 10 parents reported no allergic reaction in their children, but based on the physicians evaluation of their clinical history, their children were included in the FA group, because they have positively answered to questions referring to specific foods induced clinical manifestations. In group B, 14.1% of parents reported the onset of possible clinical allergic symptoms without prior diagnosis of FA, but the analysis of them invalidate a possible FA. In most of the cases, the allergic symptoms occurred before the age of 2 (88 cases, 12.4%), while 68.5% of children had no allergies (see Table 1).

In group A, the age of onset reported with the highest prevalence was below the age of 2 (46.0% of the children) and almost a quarter had symptoms after the age of 5. In this group, 10 parents reported no symptoms and their comment referred to skin problems during childhood that were related to any type of food. The highest prevalence was in Romanians, with 448 children, both in group A and B. Overall class distribution of the subjects illustrates the homogeneous distribution by age groups for the two levels of education: primary school (between 0 and 4th grade) and lower secondary education (between 5th and 8th grade). The prevalence of FA was 6.7% in children aged between 6 and 10, and 11.9% in children above 11 years old.

### 3.2 The prevalence of clinical features

In group A, 47 children presented skin rash with itchy lesions. In 38 of them (60.3%) the lesions were located at the ears, around the eyes and neck, ankle, popliteal and elbow area. A reduced number of children from group B had skin lesions (9.3%) (Table 2).

**Table 2:** The prevalence of food allergy clinical manifestations.

Food allergy clinical manifestation	Group A, (*n* = 63) No., %	Group B, (*n* = 645) No., %	*p*	Sensitivity%	Specificity%	PPV^a^, %	NPV^b^, %
Associated atopic dermatitis	46 (73.01%)	61 (9.45%)	<0.01	74.603	82.791	29.747	97.091
Food-induced angioedema	10 (15.87%)	15 (2.32%)^c^	<0.01	28.571	97.364	51.429	93.314
Gastrointestinal disorders	18 (28.57%)	92 (14.26%)	0.006	30.159	84.961	16.379	92.568
Wheezing episods	23 (36.50%)	137 (21.24%)	0.016	33.333	79.380	13.636	92.419
Exercise-induced wheezing	7 (11.11%)	25 (3.87%)	0.016	11.111	96.189	24.138	91.415
Asthma	14 (22.22%)	54 (8.37%)	0.001	22.222	92.558	22.581	92.415
Ithcy or runny nose without a could	34 (53.86%)	167 (25.89%)	<0.01	53.968	74.884	17.347	94.336
Sneezing, itchy eyes	24 (38.09%)	105 (16.27%)	<0.01	41.270	83.876	20.000	93.599
Allergic rhinitis	29 (46.03%)	100 (15.50%)	<0.01	46.032	85.601	24.167	94.087

^a^ PPV, positive predictive value.

^b^ NPV, negative predictive value.

^c^ Parents did not report any correlation with a specific food and tests were not performed, but the answer was positive to this question.

**Figure 1:**
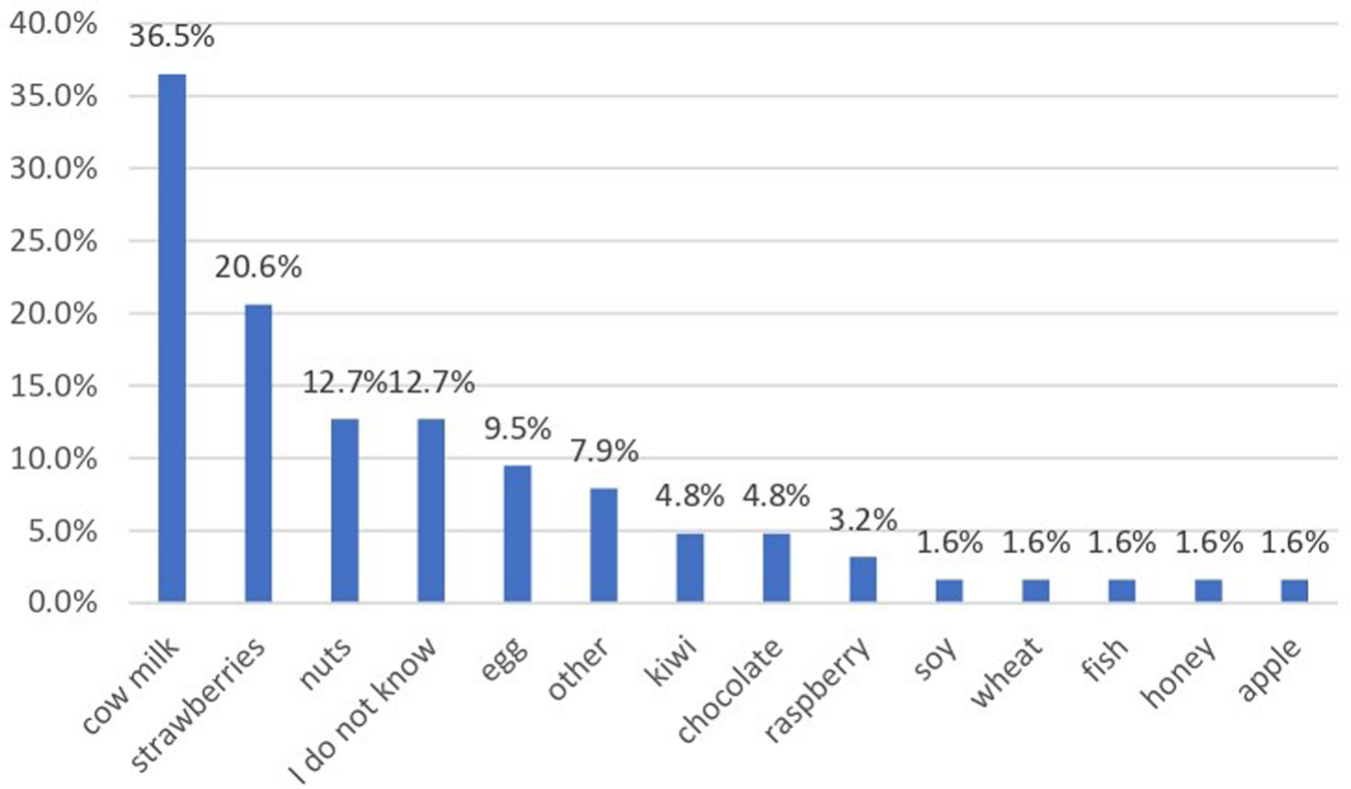
Types of food associated with allergic reactions.

The gastrointestinal disorders related to food consumption included diarrhea, with explosive or bloody stools, and abdominal pain. In group A, 30.2% of children presented such symptoms, while in group B, only 15.0% of them. The culprit foods that induced gastrointestinal symptoms were extremely variable, including cow milk and dairy products, fish and seafood, egg, different types of berries (strawberry, raspberry) grapes, eggplant, peanut and tree nuts (hazelnut, walnut) chocolate, honey, pineapple, caramel sauce, sausages, mayonnaise, cereal, and hummus (see Figure 1). Neither of these 97 children had a prior diagnosis of food allergy. The parents of 17 children from group B reported angioedema, but in the majority of the cases, the relationship with a specific food allergen was not established.

Respiratory symptoms like wheezing, itchy and/or runny nose, sneezing, itchy eyes and prior diagnosis of asthma and/or allergic rhinitis were significantly more prevalent in group A as compared to group B (Table 2).

### 3.3 Risk factors for FA

Potential risk factors for any type of allergic disease including food allergy were investigated in the questionnaire. Among these factors, we assessed family history of atopic diseases and the duration of breastfeeding Table 3. History of atopic conditions in the family and other risk factors

**Table 3:** History of atopic disorders in the family and other risk factors.

	Group A, (*n* = 63)	Group B, (*n* = 645)	*p*	Sensitivity, %	Specificity, %	PPV^a^, %	NPV^b^, %
	No. of cases, %	No. of cases, %					
Family history of allergic disorders							
Mother	36 (57.14%)	179 (27.75%)	<0.01	55.556	70.078	15.351	94.167
Father	34 (53.96%)	133 (20.62%)	<0.01	42.857	78.140	16.074	93.333
Siblings	18 (28.57%)	102 (15.81%)	<0.01	30.159	78.140	11.875	91.971
Duration of breastfeeding							
Absent	4 (6.35%)	34 (5.27%)	0.045	6.319	94.729	10.526	91.194
Less than 3 months	17 (26.98%)	125 (19.38%)	0.357	26.984	80.620	11.972	91.873
Between 4 and 6 months	12 (19.05%)	95 (14.73%)	0.004	19.048	85.271	11.215	91.514
Over 6 months	30 (47.62%)	392 (60.77%)	0.001	47.619	39.225	7.109	88.462

^a^ PPV, positive predictive value.

^b^ NPV, negative predictive value.

Regarding the other risk factor assessed, it was found that the absence of breastfeeding was similar in the two groups of children (Table 3). Breastfeeding during first 6 months of age was noticed in 60.8% of children from group B, as compared to 47.6% of children from group A, with a negative predictive value of 88.462%, but with low sensitivity and specificity.

### 3.4 Culprit foods

The questionnaire included and we analysed the main foods that might produce allergies as previously mentioned. The highest prevalence was noticed for cow milk or derivatives (yoghurt, cheese, butter) that were involved in an allergic reaction in 36.5% of cases, and strawberry in 20.6% (Figure 1). In 12.7% of cases the exact incriminated food was not established and 7.9% of parents reported different types of foods.

Spontaneous report of drug allergy was mentioned by one parent and insect-bite non-anaphylactic reaction by one parent, both children belonging to group A.

### 3.5 Severity of FA and treatment

Out of the 63 children with FA, 38.1% required presentation to the emergency department and 9.5% received epinephrine, while 34.9% received systemic (oral or intravenous) corticosteroids (Table 4). Almost all cases with FA received H1 antihistamines during the exacerbation of symptoms and after the reaction 68.3% of them had long-term exclusion diet. In group B, 5.1% of the children required emergency department presentation as reported by the parents, although they had neither consistent history nor diagnosis of FA.

**Table 4:** Allergy treatment.

	Group A, (*n* = 63) No. of cases, %	Group B, (*n* = 645) No. of cases, %	*p*
Diet restriction	43 (68.25%)	22 (3.41%)	<0.001
H1 antihistamine drugs	59 (93.65%)	224 (34.73%)	<0.001
Systemic corticosteroids	22 (34.92%)	41 (6.35%)	<0.001
Adrenaline	6 (9.52%)	8 (1.24%)	0.103
Emergency room presentation	24 (38.10%)	33 (5.12%)	<0.001

### 3.6 Teacher and medical staff awareness regarding the medical history of the schoolchildren

In what concerns the extent of information the teacher and/or the school physician have about food allergies in their children and the seriousness of the disease (severe, required diet or necessary treatment), in our study group only 33 parents from group A (52.4%) reported that they informed school authorities about these special circumstances. In group B, a statistically significant lower number of parents (38 parents, respectively 5.6%) informed the school teachers and healthcare providers on a possible suspicion of food allergy (*p* < 0.001).

Analysing the possible restrictions that children with food allergies might have during trips, parties or any other extracurricular activities with their colleagues, we noticed that only one parent from group A (1.6%) and eight parents from group B (1.2%) reported any interference.

### 3.7 Age distribution of FA symptoms

We divided the children with food allergy in group A into two age groups: young children aged between 6 and 10 years and children aged between 11 and 15 years, and we analysed the differences between them (Table 5).

**Table 5:** The two age groups in children with food allergy.

	Children aged between 6 and 10 (*n* = 27) No. (%)	Children aged between 11 and 15 (*n* = 36) No. (%)	*p*
Male subjects	12 (44.44%)	17 (47.22%)	0.890
Associated atopic dermatitis	18 (66.66%)	29 (80.55%)	0.307
Food-induced angioedema	8 (29.62%)	10 (27.78%)	0.480
Gastrointestinal disorders	9 (33.33%)	10 (27.78%)	0.520
Asthma, no. (%)	5 (18.51%)	9 (25.00%)	0.424
Allergic rhinitis, no. (%)	14 (51.85%)	15 (41.66%)	0.420

The age distribution in primary school and lower secondary education was similar, with a slightly higher prevalence in children between ages 11 and 15 (57.1%). Male subjects showed a lower percentage than female subjects in both age groups. Based on self-reported prevalence there was no significant prevalence of allergic comorbidities (atopic dermatitis, asthma or allergic rhinitis) in older children as compared to the younger group. Food-induced angioedema was reported in 29.62% of young children and 27.78% of children above the age of 11.

## 4 Discussion

The present study reports clinical characteristics and results from investigations in schoolchildren from four schools in Cluj-Napoca, Romania. To our knowledge, this is the first study that assesses food allergy in Romania, mainly in the pediatric population. During the early 1990s, several epidemiological studies on allergic diseases were carried out in Romania (7). The prevalence of asthma, eczema and allergic rhinitis were assessed using a standardized study protocol, designed by the International Study on Asthma and Allergic Diseases in Children (ISAAC) (7, 8). This questionnaire did not include specific questions about food allergy, therefore the researchers did not have reliable data to compare the extent of FA in our geographical area.

A decade ago, the incidence of allergic diseases was characterized as a wave of the allergic epidemic, which mainly affected infants and preschool children (3). Based on the lack of epidemiological data between the ISAAC study and the time point in 2010, the surveys on the prevalence of FA proved the increasing trend of all allergies, including FA. In a more recent review, Spolidoro et al. commented that although the frequency of FA in Europe seems to have an increasing trend, there are still not enough revised data (9). Between 2000 and 2012, the lifetime prevalence was 5.9%, while during the past decade the prevalence increased almost three times, up to 14.9% during 2012 and 2021. Their analysis estimates that the current prevalence of any FA during a lifetime in children is 18.7%, with a point prevalence of reported FA of 14.2%. The review included 110 studies and showed that any FA prevalence was higher in Eastern and Northern Europe as compared with Southern and Western Europe. The authors emphasized that there is a very limited number of studies from Eastern Europe (9). None of these studies reflected the prevalence of FA in Romania.

We compared the data in our study, conducted in Cluj-Napoca (Romania), with current data on prior prevalence of self-reported FA in other European countries (1) and worldwide (2). The overall prevalence of food allergy in our study group was 8.89%. The prevalence in our study is lower than the previous prevalence of self-reported FA to different foods in Europe. The prevalence varied in European countries between 13.1% in Athens (Greece), 16.3% in Zurich (Switzerland), 16.7% in Reykjavik (Iceland), 17.1% in Utrecht (The Netherlands), 17.9% in Madrid (Spain), 19.7% in Sofia (Bulgaria), up to significantly higher percentages of 43.4% in Lodz (Poland) and 47.5% in Vilnius (Lithuania) (1). The percentages reported in other countries were lower, and similar to our overall prevalence, when data were addressed only to priority foods.

Over 160 foods are incriminated in food allergy (2). The present study analyzed the answers to 12 foods and the authors offered parents the opportunity to add other food allergens that triggered the reaction in their child. Among the main allergenic foods, a higher prevalence was recorded for milk or derivatives (36.5%), different types of nuts (12.7%) and hen egg (9.5%), with a very low prevalence for wheat or cereals, fish, seafood and soybeans. Out of the other foods, the most common were strawberries (20.6%), while 12.7% of parents could not report a certain food involved in the FA. In a recent survey, Messina M et al. refer to priority foods as the Big 8, as classified by the Food Allergen Labeling and Consumer Protection Act (FALCPA) (10). The origin of this classification of foods relies on the prevalence of allergic reactions to different type of foods as well as clinical evidence of severe reaction to food, including fatal anaphylactic shock. Based on data from 5 surveys in large population samples on the prevalence of self-reported FA in children, the authors noticed that the prevalence of soybean allergy is lower than the other 7 major allergen. The Japanese list includes only 7 food allergens for mandatory labeling, soy being excluded. On the other hand, sesame allergy seems to be increasing, therefore sesame is a potential candidate for the Big 8 list (10). A list of 14 major food allergens is currently being discussed in the European Union. The observed differences could be explained also by different dietary particularities. In Romania fish and seafood is not a common foods included in many diet, so the exposure to these ones is reduced. The same observation is also available for soybean.

We noticed an unexpected high prevalence of strawberry allergy in our study group. Several authors report strawberry allergy, with prevalence values between 0.3% and 9.2%, but with a very high prevalence of this allergy among severe reactions, up to 13.2% (11–14). It is relevant to emphasize that strawberry allergy can be a cross-reactions with pollen allergy. In Romania grass pollen season coincides with strawberry season and many parents consider that an acute urticarial is a consequence of food ingestion rather than a secondary reaction to pollen exposure.

The estimated prevalence of FA in children from the United States in 2005 was 6.0% for any food, 2.5% for milk, 1.3% for egg, 0.8% for peanut, and 0.1% for fish (10). In a study performed as a random-digital-telephone survey with 20,686 individuals, both children and adults, the prevalence of self-reported FA in children from the United States was 6.53% for any food, 1.94% for milk, 0.64% for egg, 1.16% for peanut, and 0.43% for fish (15). In contrast, children from Canada included in Messina M. review showed higher prevalence than those in the United Stated, with 7.14% FA for any food, 2.23% for milk, 1.77% for peanut, 1.23% for egg, but lower prevalence rates of 0.18% for fish (10, 15). These last two surveys were carried out during 2007–2010 (15) and 2008–2009 (16).

This variable prevalence is depending on the method of data collection and supplementary investigations needed to confirm the positive diagnosis of FA. In the present study, the authors analyzed only the lifetime self-reported prevalence of FA among children, referring also to the main foods analyzed also in the aforementioned research. Each type of analysis have benefits and limits. Self-reported prevalence of FA gives a rapid estimation of possible patients with FA, but for an accurate positive diagnosis, the confirmation is needed.

There are differences in the prevalence of FA in different age groups. In the review published in 2013, the authors reported a higher prevalence in younger children, below the age of 5 or even during infancy, as compared with children above the age of 5 (3). In our study, the overall prevalence of self-reported FA in children aged between 6 and 10 was 6.67%, while in older children, above the age of 11, the prevalence of FA was reported in 11.88% of them. Some other studies also showed that in older children, mainly above the age of 14, the prevalence of FA is lower than in the younger group or when compared to the overall prevalence in children of all ages (4, 10). Previous studies showed that self reported lifetime prevalence of FA is higher in younger than in older children. This could be explained also by the fact that some allergies (e.g., milk or egg allergies) may have spontaneous resolutions after 5 years old. The present study did not included children of 5 years old because parents of schoolchildren completed the questionnaire and in Romania the minimal age for primary school is 6 years old. But the same tendency is maintained in the present research.

Regarding potential risk factors for FA, our data showed a significantly higher prevalence of family history of allergic diseases in group A, as compared to no family history in 55.81% of the children in group B. Breastfeeding for a period of less than 3 months and between 4 and 6 months was similar in both groups, as well as the absence of breastfeeding. The only significant difference was regarding the duration of breastfeeding for more than 6 months, a higher rate being reported in children from group B (60.77%) as compared to children in group A (47.62%), with a negative predictive value of 88.462%. Current recommendations for the duration of breastfeeding is a minimum of 4 months according to the World Health Organization, respectively for at least 6 months according to the EAACI (European Academy of Allergy and Clinical Immunology). The data published does not yet provide consistent evidence about the beneficial and protective role of prolonged breastfeeding (2). Recent data suggest that early exposure of infants to various food allergens could induce tolerance and improve the maturation of the mucosal immune system (2).

The authors assessed the association of other allergic diseases in our study group. Children with FA had significantly higher rates of atopic dermatitis, allergic rhinitis and allergic asthma as compared to children from group B. When the two age groups of children with FA were analysed, the prevalence of these comorbidities was similar for both children between 6 and 10 years of age and for those older than 11. In a recent analysis on 3,233 individuals, Peters et al. stratified FA in infants in different phenotypes and noticed a correlation of early onset of FA with lower pulmonary function tests after the age of 6 (17). Even if the children presented transient egg or peanut allergy, their FEV1 had lower values. Compared to the general population, food allergy, and in particular egg allergy, correlates with a significantly higher prevalence of atopic dermatitis, asthma or eosinophilic esophagitis (18). The prevalence of egg allergy was reported by Samady et al. in a complex survey on 38,408 children, 1.3% in children below the age of 5, while the overall prevalence of egg allergy in children all ages was of 0.9% (18). Additional to atopic dermatitis, “food-protein induced protein losing enteropathy” (FPIPLE) was described in children with allergic reaction to egg, cow milk and nuts (19).

The main limitation of our study is the lack of proof that FA is based on either demonstration of specific IgE and an oral food challenge test (OFC) or double-blind placebo-controlled food challenge (DBPCFC). The parents reported FA based on convincing clinical history and prior diagnosis of FA established by a physician, either paediatrician or allergist. This could lead to the overestimation of FA, with inclusion of both IgE-mediated reaction and other reactivity to food, like food intolerance, food toxicity or non-IgE mediated reactions. A minority of the parents added in their comments that they underwent either skin prick-tests and/or specific serum IgE for foods. Since we did not formulate a question on diagnostic tools, we did not report these data. In fact, in a study carried out by Lyons SA, the applied protocol had three phases: the first one analysed self-reported FA, the second phase included food-sensitized patients based on specific serum IgE, and the third investigated the patients through DBPCFC (1). The authors defined possible FA when patients with positive tests had concurrent symptoms. The prevalence of probable FA dropped to 1.9% from 5.6% children across Europe, with a match between self-reported FA and food-sensitization of about 17.2%, depending on the various foods tested. The best match was proven for lentils, apple and hazelnut (between 37% and 46%) and poorer correlation for different seeds or corn. Cow milk had a match of 8% in this study and hen egg 15.8% (1). The authors reported that very few patients in their multinational study group agreed to take part in phase III (18 of 16,935 subjects). The lack of exact data regarding real confirmed FA based on gold standard for the diagnosis is a recognised as bias in other studies. The DBPCFC involves risks that require the test to be performed in specialized centres (20, 21). The overestimation of the prevalence of FA can also be due to the increased awareness in recent years, the situations in which investigation is requested through skin tests or specific IgE being more frequent. Component-Resolved-Diagnosis for the identification of IgE is a diagnostic method recently introduced in the evaluation of allergic patients. However, its high costs limit access to this investigation (20). The great majority of studies on prevalence are based on questionnaires with self-reported FA or parent-reported FA. Even a physician-diagnosed allergy is not always reported in prevalence studies. In a large cross-sectional survey on egg allergy, 27.8% of the participants did not have physician-diagnosed allergy (18, 22).

The most recent review on the frequency of FA in Europe summarized self-reported FA in 20% of the children, the sensitization proven with skin prick tests in 6% of them and with IgE in 17%, while food challenge-verified FA was 0.8% (9). The most commonly used food challenge was OFC, as compared with DBPCFC. The authors emphasized the significance of a convincing clinical history, and the fact that the recent studies did not include any of the challenge tests in the assessment of the prevalence of FA.

The burden of FA is even greater as the number of specialists is reduced and the availability of adrenaline auto-injectors is still low. In Romania, these children are referred to either the pediatrician, or to the allergist, since in Romania there is no distinct medical specialty of pediatric allergology and immunology. Although the most severe cases are primarily diagnosed in the Emergency Department, the diagnostic work-up, the training in self-administration of adrenaline, and the role of other therapeutic options are part of a subsequent evaluation. Adrenaline auto-injectors (AAI) are available in Romania, but they are not reimbursed by the healthcare system. In our study group, the number of children that were addressed to the Emergency Department due to an acute event was 24 (38.10%) in group A and 33 (5.12%) in group B, but only 14 children received adrenaline during acute episodes. A higher number of children received systemic steroids (34.92% from group A and 6.35% from group B) and almost all children in group A (93.65%) were treated with H1 antihistamines for acute symptoms. In a recent cross-sectional study on FA in children from the US, there were 47.7% FA-related Emergency Department visits in Hispanic individuals and 45.4% in African-American individuals (10). Other studies have reported a higher Emergency Department presentation rate of children with egg allergy, as 21.1% of the children with egg allergy report severe reactions, compared to those with allergic reactions to other foods (18, 22). The use of AAI was 20.9% in Caucasian children and even higher, up to 23.6% and 24.6% in African-American and Hispanic children. The authors analyzed the prescription of AAI and the rates were above 25% in all races (23). The strict requirement for training in order to prevent severe FA events, mainly the use of AAI, was pointed out in a recent review (24). Food-induced anaphylaxis, including fatal reactions, have demonstrated an increasing prevalence during recent decades (22, 25). This aspect leads to increasing concern for families as well as the need for greater awareness for policymakers.

In the present study, the authors assessed the incidence of severe FA, teacher and school physician awareness about the history and therapy of FA of schoolchildren, as well as the parents' training on the use of adrenaline. Only 52.38% of parents informed the teacher and the school physician on their child's history of FA. Although 38.10% of children had prior visits to the Emergency Department and 28.57% had proven food induced angioedema, the number of patients who received adrenaline during a severe episode was of only 9.52% of total cases with FA. The low percentage of parents who reported the health issues to the teaching staff and to the school physician has no reasonable explanation. The number of children with severe FA in primary school was double compared to children in lower secondary education, aged above 11, and this is an aggravating factor for the risk of accidental exposure during school time. This is an alarm for a better training of children with FA and their parents, for the awareness on the potential risks and for the crucial role of informing the teaching and medical staff in the school. Surprisingly, the authors noticed that parents with children with FA declared that their children have no issues regarding extracurricular activities with their colleagues, since only up to 1.59% of them reported interference with social activities like parties or trips.

The management of severe allergic reactions in the community is a constant topic for allergists worldwide. A decade ago, Food Allergy and Anaphylaxis Guidelines were published by the European Academy of Allergy and Clinical Immunology (EAACI) (26). The main purpose of this guideline was, on one hand, the high percentage of severe reactions induced by food, and on the other hand, the fact that these reactions usually take place in the community (kindergarten, school, restaurants, playgrounds, etc.). Parents have the responsibility to take the appropriate measures for their child when recognizing potentially risky circumstances, avoiding specific allergens and training to use emergency medication, like adrenaline auto-injectors (AAI). Food allergy and its potentially severe course is little known by teachers, who have poor knowledge about anaphylaxis and, furthermore, about the appropriate management. The fact that parents do not inform the school about the student's allergy increases the risk of severe and potentially fatal reactions. In 2020, a questionnaire-based assessment was conducted on raising awareness to allergic pupils in schools, training of school staff and parents on the correct treatment of allergies (27). The information about the number of children with severe FA was correct, but the preparedness for its management was poor. A high percentage of schools (81%) expressed the need for further training. A questionnaire-based study on the preparedness of school teachers in Greece regarding FA has recently been published (28). The results confirm the lack of knowledge in teachers and other school staff members, as well as school principals, both on the symptoms and on the use of adrenaline auto-injectors. Similar data were published in Saudi Arabia (29) and Italy (30). Artificial intelligence (AI) could be an important tool for education, addressing to both parents and teachers. Using AI the parents could learn how to use the auto-injector, while teachers may learn how to detect a characteristic symptom for allergic reactions. A recent study performed in Romania showed that AI had a good acceptance among caregivers of children (31).

## 5 Conclusions

Lifetime self-reported prevalence of FA was 8.89% in a cohort of schoolchildren, with lower values in those between 6 and 10 years old. Self-reported anaphylaxis was mentioned in 28.57% of children with FA. Family history of allergic diseases was correlated with a higher risk for FA. Breastfeeding was not found to be a significant protective factor to FA development. The use of AAI was reported in very few children and parents informed the teaching and medical staff in schools on their child's allergy in a very low percentage. These aspects are strong recommendation for further educational programs for children and parents of children with FA, and also for teachers and school staff. These data offer a new perspective regarding the perception of FA in a country with a relative recent major change in lifestyle that has impacted both nutritional and allergic behavior in children and young adults.

## Data Availability

The raw data supporting the conclusions of this article will be made available by the authors, without undue reservation.
